# Route of infection alters virulence of neonatal septicemia *Escherichia coli* clinical isolates

**DOI:** 10.1371/journal.pone.0189032

**Published:** 2017-12-13

**Authors:** Bryan K. Cole, Edgar Scott, Marko Ilikj, David Bard, Darrin R. Akins, David W. Dyer, Susana Chavez-Bueno

**Affiliations:** 1 Department of Pediatrics, University of Oklahoma Health Sciences Center, Oklahoma City, Oklahoma, United States of America; 2 Department of Microbiology and Immunology, University of Oklahoma Health Sciences Center, Oklahoma City, Oklahoma, United States of America; Institut Pasteur, FRANCE

## Abstract

*Escherichia coli* is the leading cause of Gram-negative neonatal septicemia in the United States. Invasion and passage across the neonatal gut after ingestion of maternal *E*. *coli* strains produce bacteremia. In this study, we compared the virulence properties of the neonatal *E*. *coli* bacteremia clinical isolate SCB34 with the archetypal neonatal *E*. *coli* meningitis strain RS218. Whole-genome sequencing data was used to compare the protein coding sequences among these clinical isolates and 33 other representative *E*. *coli* strains. Oral inoculation of newborn animals with either strain produced septicemia, whereas intraperitoneal injection caused septicemia only in pups infected with RS218 but not in those injected with SCB34. In addition to being virulent only through the oral route, SCB34 demonstrated significantly greater invasion and transcytosis of polarized intestinal epithelial cells *in vitro* as compared to RS218. Protein coding sequences comparisons highlighted the presence of known virulence factors that are shared among several of these isolates, and revealed the existence of proteins exclusively encoded in SCB34, many of which remain uncharacterized. Our study demonstrates that oral acquisition is crucial for the virulence properties of the neonatal bacteremia clinical isolate SCB34. This characteristic, along with its enhanced ability to invade and transcytose intestinal epithelium are likely determined by the specific virulence factors that predominate in this strain.

## Introduction

*Escherichia coli* is the most common Gram-negative bacterium causing neonatal sepsis in the United States. *E*. *coli* now surpasses group B streptococcus as the most common cause of neonatal bacteremia in premature newborns and in otherwise normal febrile infants [1–3]. Mortality in newborns with *E*. *coli* bacteremia is as high as 40% [4], and meningitis is often a devastating consequence associated with severe neurodevelopmental disabilities in survivors [5]. There is currently no vaccine or any other preventive strategy to control neonatal *E*. *coli* sepsis. The incidence of this disease continues to increase and rising antibiotic resistance rates are a public health concern as well since this drastically limits treatment options [6].

The pathogenesis of *E*. *coli* bacteremia in newborns is not well understood. Bacterial passage across the neonatal gut after ingestion of maternal *E*. *coli* strains around the time of delivery likely is an early step in pathogenesis, as it has been demonstrated in animal models [7, 8]. Neonatal *E*. *coli* strains invade and transcytose intestinal epithelial cells prior to septicemia [9, 10]. Although several *E*. *coli* virulence factors important for invasion of the blood-brain barrier have been characterized, including the K1 capsule, OmpA, IbeA, Cnf1, and NlpI [11–14], those that contribute to intestinal translocation and neonatal septicemia prior to, or in the absence of meningitis, have not been studied in detail.

We have recently compared the *in vitro* intestinal invasion ability of several neonatal *E*. *coli* bacteremia isolates and demonstrated that some strains invaded intestinal epithelium significantly better than others [15]. Strains that belonged to the increasingly prevalent multi-locus sequence type (ST) 131 were significantly more invasive than those within other STs. Clinical isolate SCB34 was identified among the high invasive ST131 group. Here, we have focused on this contemporary, non-K1 strain and investigated its virulence in newborn animals, its ability to transcytose across polarized intestinal epithelium, and compared its genome to the archetypal meningitic strain RS218. Our results provide evidence that the virulence of SCB34 is dependent on the oral route of inoculation. This is in contrast to RS218, which is fully virulent following introduction into neonatal rats by either oral or intraperitoneal inoculation. Interestingly, SCB34 also was found to be significantly more efficient in traversing polarized intestinal epithelium by transcytosis than strain RS218. Finally, a comparison of the genomes between SCB34 and RS218 revealed the presence of candidate virulence factors in SCB34 not found in RS218 that may be related to the specific virulence properties and increased invasiveness after oral inoculation of this unique clinical isolate.

## Materials and methods

### *E*. *coli* strains and intestinal epithelial cells

SCB34 is a neonatal bacteremia *E*. *coli* clinical isolate recovered from a septic newborn as previously described, which was formerly referred to as strain 10 in a prior study [15]. SCB34 is an ST 131 *H30* strain that demonstrated resistance to multiple antibiotics including ciprofloxacin and gentamicin, and a high invasion phenotype [16]. RS218 is an archetypal *E*. *coli* K1 strain that was isolated from the cerebrospinal fluid of a neonate with meningitis [17]. *E*. *coli* laboratory strain DH5α has been described [18]. T84 intestinal epithelial cells were obtained from the American Type Culture Collection (cell line CCL-248). T84 cells were maintained in a 1:1 mixture of Dulbecco's modified Eagle's medium and Ham's F-12 medium (Gibco, Invitrogen; Carlsbad, CA) and 100 U/ml penicillin, 100 μg/ml streptomycin supplemented with 5% (v/v) fetal bovine serum, and incubated at 37°C, 5% CO_2_. Upon full confluency, T84 cells develop tight junctions and differentiate into polarized monolayers when grown *in vitro*. The development of T84 epithelial polarization was determined by an increase of transepithelial electrical resistance as described [19].

### Newborn rat models of bacteremia

Two distinct models were used to compare the virulence properties of SCB34 and RS218 in newborn animals. DH5α was used as a control nonpathogenic strain. Outbred pregnant Sprague-Dawley rats with timed conception (Charles River Laboratories International Inc., Wilmington, MA) gave birth 7–9 days after arrival in our vivarium. For oral inoculation experiments, newborn (22 to 26 hour-old pups) were randomly cross-fostered among litters prior to receiving each 10^5^ CFU of mid-log bacteria grown in lysogeny broth (LB) medium. The inoculum was mixed in a 10 μL aliquot of PBS/0.1% gelatin and was administered orally with a sterile micropipette in a nontraumatic manner, allowing the pup to actively suck and swallow. Pups were assessed twice daily for signs of illness, and for up to seven days post-infection (p.i.). Humane endpoints were used in these animal experiments, and pups were monitored for possible signs of rapid progression of bacteremia which included: Labored breathing, hypothermia to touch, lethargy, gross weight loss or poor feeding, immobility/loss of righting reflex, or severe tremor. If any of these signs were present, pups were humanely euthanized prior to 7 days. Any mortality was recorded throughout the experiment, and on day 7 p.i. blood was collected to determine the presence of bacteremia. For intraperitoneal experiments, pups of similar age as above were injected with 10^2^ CFU of each strain intraperitoneally (IP). The inoculum was grown in identical fashion and was mixed in a 50 μL PBS/0.1% gelatin aliquot that was injected IP. Identical humane endpoints were also used for the IP animal experiments. The pups were observed twice daily for up to 3 days p.i., and 50 μL of blood was then collected for determination of bacteremia prior to euthanasia after this observation period. In both models, groups of 10 pups per strain were tested. All animals were anesthetized with inhaled isoflurane prior to blood collection to minimize suffering. Humane euthanasia was performed by CO_2_ inhalation. No unexpected deaths occurred, as all deaths were attributed to bacteremia. The proportion of mortality or bacteremia in each group was compared using the chi-square or Fisher’s test as appropriate; a *p* value <0.05 was considered significant. All animal experiments were performed with the approval and in strict accordance with the recommendations of the University of Oklahoma Institutional Animal Care and Use Committee (IACUC), under protocol number 15-099-I.

### Intestinal epithelial cell invasion assays

To confirm the ability of SCB34 and RS218 to invade intestinal epithelium *in vitro*, invasion assays were performed similarly to previously described [15], using a modified gentamicin protection assay. The modifications were done taking into account SCB34’s resistance to gentamicin but susceptibility to amikacin, and to improve the efficiency of our methods. Briefly, T84 intestinal epithelial cells were grown to 75 to 80% confluence in 24-well tissue culture plates. Prior to infection, antibiotic-free tissue culture media was substituted overnight. Each *E*. *coli* strain was grown to mid-logarithmic phase in tissue culture media (TCM) and 10^6^ CFU of each *E*. *coli* isolate were used to infect triplicate wells for an approximate multiplicity of infection of 10 per well. After centrifugation at 800 x*g* for 5 minutes at 4°C, the infected epithelial monolayers were incubated for 1 hour at 37°C, 5% CO2, to allow bacteria to invade. The monolayers were then washed and treated with amikacin at a concentration of 200 μg/mL (Sigma-Aldrich; St. Louis, MO) for two additional hours to kill extracellular bacteria. After antibiotic treatment, the T84 cells were washed, lysed with 0.1% Triton X-100, and the recovered intracellular bacteria were quantified. The percent invasion was calculated as follows: (CFU recovered/CFU inoculated) x 100. Mean invasion percent was compared among the strains using one-way ANOVA comparisons; a *p* value <0.05 was considered significant.

### *In vitro* bacterial transcytosis assay

A transcytosis assay was used to assess the ability of the clinical isolates to translocate across polarized T84 cells. T84 cells (10^4^, passage 7–10) were seeded onto 0.3-cm^2^ growth area semipermeable filter supports with 3.0 μm pores (Corning; Corning, NY). Cells were cultured for 7–14 days until becoming fully confluent and polarized, showing a transepithelial electrical resistance (TEER) of ≥ 1000 Ohm·cm^2^ measured by a voltmeter and companion electrodes (World Precision Instruments; Sarasota, FL) [20]. Inserts with polarized T84 cells were transferred to antibiotic-free TCM overnight and then were infected on the apical side with 10^5^ mid-log CFU *E*. *coli* per insert mixed in antibiotic-free TCM containing 1 mg/mL of fluorescein isothiocyanate (FITC)-dextran 4000Da (FD4) (Sigma-Aldrich; St. Louis, MO) [21]. Non-infected inserts were included as additional controls. The inserts were maintained at 37°C, 5% CO2 throughout the experiment. At 30-minute intervals, the inserts were transferred to collecting wells containing fresh sterile antibiotic-free TCM, and the collecting well TCM from the previous time point was retrieved for CFU quantification on LB agar plates. After the last time point at 6 hours p.i., FD4 concentrations were also determined using the Tecan Infinite® 200 PRO Microplate Fluorescence Reader (Tecan; Männedorf, Switzerland). FD4 measurements allowed us to compare the permeability to this small molecule of the polarized infected T84 cells to that of non-infected monolayers. Baseline and post-infection TEER values were also obtained. Each strain was tested in groups of 5–6 inserts per experiment, in 3 separate experiments. The amount of transcytosed CFU after infection with each strain was analyzed using a mixed-distribution Bayesian model to account for the presence of zeroes, and the variable number of inserts per experiment. An adapted repeated measures, zero-inflation Poisson (ZIP) model [22] fit the data best. The initial, structural zeroes from the ZIP model were analyzed using a discrete-time logistic regression model predicting time until first translocation, and all subsequent well outputs were analyzed using a generalized linear model predicting piece-wise growth for a Poisson outcome. Models were fit using MCMC estimation in WinBUGS [23]. A log-transformation was applied to sum of one plus each data observation. Bayesian 95% confidence intervals were used to determine statistical significance (i.e., intervals for significant contrasts did not contain zero).

### Growth curve comparisons in lysogeny broth (LB) and tissue culture media

Growth was compared between SCB34 and RS218 in the two different media that were used in the *in vitro* and *in vivo* experiments described above. A single colony from each strain grown overnight on LB agar was used to inoculate 5 mL of either LB or TCM that were incubated overnight at 37°C with shaking at 250 rpm. A 1:100 dilution of these overnight cultures was incubated in either LB or TCM for 2 hours at 37°C, 250 rpm. The two-hour cultures were pelleted by centrifugation and resuspended to an optical density (OD) at 605 nm of 0.7 using LB or TCM as appropriate before inoculating fresh LB or TCM (0.1% v/v). Growth curves were performed in 300 μL volumes with five replicates for each growth condition in each individual experiment. OD measurements were taken at 600 nm at thirty-minute intervals with the Bioscreen C Microbiology Reader (Oy Growth Curves AB Ltd.; Helsinki, Finland) set to incubate at 37°C with constant shaking (machine setting “low”). In addition to OD measurements, growth curves in either LB or TCM were performed for each isolate by CFU plate quantification at 30-min intervals. One-way repeated measures ANOVA was used to compare OD values between the strains. A *p* value <0.05 was considered significant.

### Sequence analysis and comparison among *E*. *coli* isolates

To identify and compare the gene content of SCB34 and RS218, we first performed whole-genome sequencing of these two isolates on an Illumina MiSeq using a 250-bp paired-end library. The paired end reads were assembled *de novo* using the A5 assembly pipeline and annotation was performed using RAST or the National Center for Biotechnology Information (NCBI) Prokaryotic Genomes Annotation Pipeline, respectively [16, 24]. We then compiled a database of SCB34, RS218 and the annotated genomes from 33 phylogenetically diverse strains representative of all *E*. *coli* strains deposited in GenBank. Clusters of putative orthologous proteins were generated for all strains examined using CD-hit [25]. The sequence identity threshold utilized was 80% across 80% of the total protein length while all other parameters remained at the default values on CD-hit. From the CD-hit output, a database was generated that contained 22,084 clusters including between 1 and 35 genomes per cluster. Using python scripts, the orthologous protein cluster results from CD-hit were organized into a genome versus protein cluster tables in which the presence or absence of an ortholog in a given genome is identified with either a 1 or 0, respectively. Heatmaps were created from comparison tables using the gplots package in R (version 2.17.0 [http://CRAN.R-project.org/package=gplots]), employing hierarchical clustering to compare rows and columns and to construct the dendrograms. The list of the 22,438 clusters that were included in the CD-hit output, and their respective accession numbers are detailed in S1 Table.

Cluster sequences identified with CD-hit were manually curated by searching the NCBI Microbial Genomes database using the Basic Local Assignment Search Tool (BLAST) and querying all representative genomes, optimizing for highly similar sequences (Megablast).

## Results

### Clinical *E*. *coli* isolates SCB34 and RS218 display unique oral and intraperitoneal virulence phenotypes

**Fig 1:**
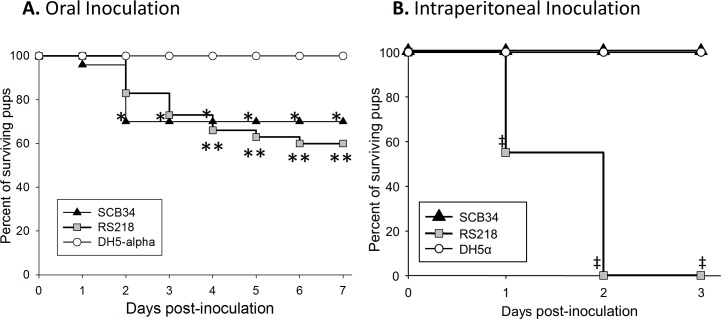
Virulence in newborn rats after inoculation with neonatal *E*. *coli* isolates SCB34 or RS218. **1A.** Animals were inoculated orally and survival post-inoculation is shown (data from three separate experiments, n = 10 animals per group, per experiment). *p<0.*05 SCB34 vs*. *DH5α*, **p<0.*03 RS218 vs*. *DH5α*. **1B.** Survival after intraperitoneal inoculation (data from two separate experiments, n = 10 animals per group, per experiment). *‡p <* .*001 RS218 vs*. *SCB34 or RS218 vs*. *DH5α*.

To compare the virulence of SCB34 and RS218 in newborn animals, individual groups of neonatal rats were inoculated orally with either clinical isolate, or the nonpathogenic DH5α strain (Fig 1). Oral inoculation with SCB34 or RS218 resulted in comparable mortality of 30% and 40%, respectively, which was significantly higher than in animals inoculated with DH5α, which demonstrated no illness (Fig 1A). Among surviving animals on day 7 after oral inoculation, bacteremia was present in 14% and 39% of those infected with SCB34 or RS218, respectively (*p* = ns,S7 Table), and in none infected orally with DH5α. In contrast, IP infection of neonatal pups with SCB34 or DH5α produced no mortality, whereas IP inoculation with RS218 killed all animals within two days (Fig 1B). Bacteremia in survivors 3 days after IP inoculation with SCB34 or DH5α was not significantly different (S7 Table).

### Invasion of intestinal epithelial cells in vitro by neonatal *E*. *coli* clinical isolates

**Fig 2:**
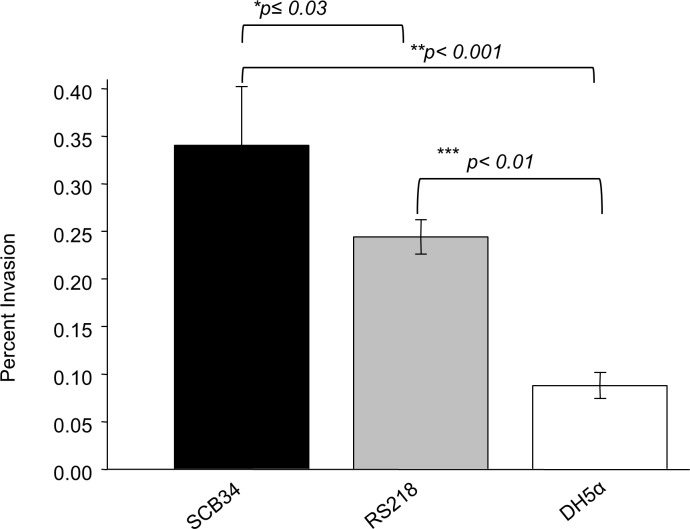
Invasion of intestinal T84 epithelial cells by SCB34 and RS218. The nonpathogenic laboratory strain DH5α was used as control. SCB34 demonstrated the highest invasion ability among the three strains. *SCB34 *vs*. RS218 (p≤0.03); **SCB34 *vs*. and DH5α (p<0.001); and ***RS218 *vs*. DH5α (p<0.01).

We next examined the invasion capacity of SCB34 as compared to RS218. The non-pathogenic laboratory strain DH5α was used as a negative control. In contrast to the results of the virulence assays above, strain SCB34 was significantly more invasive compared to the pathogenic RS218 strain (p≤0.03) and also more invasive than the DH5α laboratory strain (p<0.001) (Fig 2). As expected, RS218 was more invasive than the nonpathogenic DH5α (p<0.01).

### Transcytosis comparisons of neonatal *E*. *coli* clinical isolates across polarized intestinal epithelial cells

**Fig 3:**
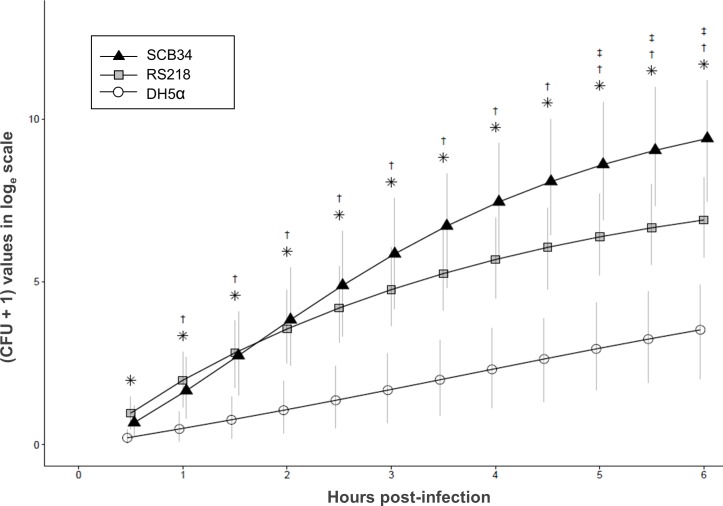
Transcytosis of *E*. *coli* clinical isolates across polarized intestinal epithelial cells. *p<0.05 RS218 vs. DH5−α; † p<0.05 SCB34 vs. DH5−α; ‡ p<0.05 SCB34 vs. RS218. Model−calculated mean of cumulative log_e_ (CFU+1) values over time are shown. Error bars reflect 95% Bayesian confidence intervals. Points were jittered to improve clarity (combined data from three separate experiments).

SCB34 and RS218 were compared regarding their *in vitro* ability to transcytose polarized intestinal epithelium. Polarized T84 cells grown on permeable inserts were inoculated with both isolates and the number of bacteria recovered in the collecting wells was compared over time. Fig 3 shows the calculated cumulative number of CFU at each 30-min time point after apical inoculation of polarized T84 cells with each strain. Significantly greater numbers of cumulative CFU were recovered from collecting wells of polarized epithelia infected with RS218 or SCB34, starting at 0.5 and 1 hrs. p.i., respectively, as compared to the noninfectious control strain DH5α. When comparing SCB34 and RS218, significantly greater cumulative amounts of CFU transcytosed from SCB34 infected inserts starting at 5 hrs. p.i.. The amount of FD4 recovered at the end of the 6 h incubation in the collecting wells of inserts infected with each *E*. *coli* strain was no different compared to non-infected inserts, which indicates that the paracellular permeability of the polarized epithelium did not increase after infection. These results suggest that the bacteria recovered from all inserts passed across the polarized intestinal cell barrier via the transcellular (not the paracellular) route. The integrity of the polarized monolayers throughout the infection process was further corroborated by measuring TEER values at the end of the 6 h incubation, which were maintained through this time point p.i..

### Growth curves of neonatal *E*. *coli* isolates

**Fig 4:**
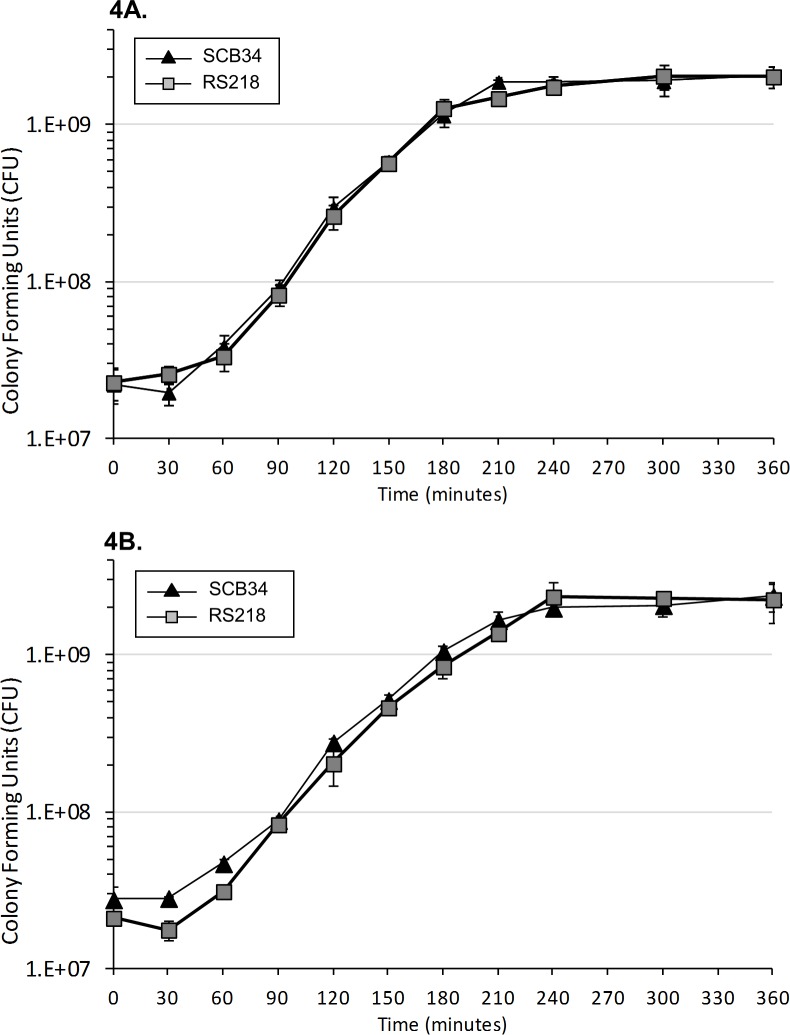
Growth curves of neonatal *E*. *coli* clinical isolates RS218 and SCB34. Growth curves performed in (4A) LB media and (4B) tissue culture media (TCM) by plate counts. Each plot shows mean CFU values in triplicate samples per time point of each individual clinical isolates. Error bars represent one standard deviation.

The animal experiments, and tissue culture assays were performed with bacteria from mid-logarithmic phase cultivated in either LB or TCM, respectively. We confirmed that the rate and extent of growth of SCB34 and RS218 was indistinguishable, when measured either by viable count (Fig 4A and 4B) or by optical density, indicating that the differences observed in invasion, virulence, and transcytosis were not likely the result of differences in growth rates among the strains examined.

### Whole-genome comparisons between SCB34, RS218 and other representative *E*. *coli* isolates

**Fig 5:**
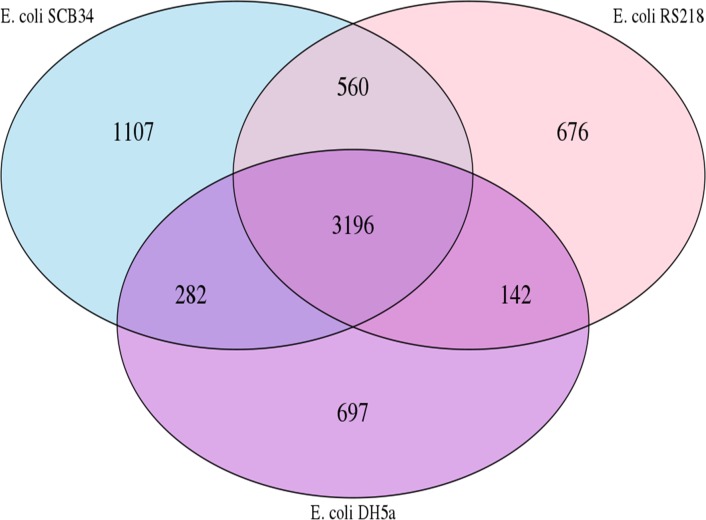
Comparison of gene distribution among SCB34, RS218, and DH5α. The Venn diagram shows genes that are common or unique genes in each isolate. The unique proteins have been determined by comparison between the 3 strains.

To begin to understand the basis for the phenotypic differences between SCB34 and RS218, we compared the protein coding sequences of these two strains [16, 24] using DH5α as a nonpathogen for comparison. The rationale for this comparison is that the protein coding capacity within each genome largely represents the biologically functional capacity for each strain (regulatory RNAs and non-coding regulatory sequences notwithstanding). Fig 5 displays a comparison of all protein coding sequences found in SCB34, RS218, and DH5α in a Venn diagram. Surprisingly, the number of protein coding sequences unique to strain SCB34 (1,107) was almost twice the number unique to either RS218 (676) or DH5α (697), and so this comparison suggested a greater functional complexity of SCB34 compared to the archetypal neonatal isolate RS218.

**Fig 6:**
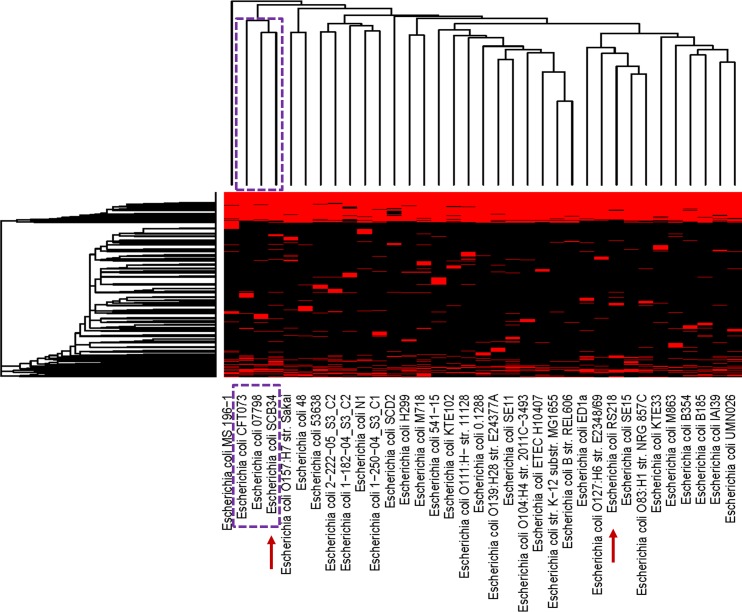
Heat map comparing clusters of genes present (red) or absent (black) in each *E*. *coli* genome. SCB34 and RS218 are highlighted by the arrows, among 33 additional representative *E*. *coli* strains. The dotted-line boxes highlight the clade that includes SCB34, and the names of the two additional *E*. *coli* strains with the most similar protein-coding sequences within the clade. A hierarchical clustering of the genes found in all genomes is found at the left of the heat map, while a tree based on hierarchical clustering of genomes is shown at the top of the heat map.

Given the large number of differences observed among these three strains, we next compared the protein coding capacity of SCB34 to that of a representative group of 33 *E*. *coli* strains with the goal to focus on the unique biological potential of strain SCB34. The list of all strains and their respective phenotype included in this comparison is included in S4 Table. Informative clusters of orthologous proteins genes (COGs), excluding clusters unique to each genome and core clusters common to all genomes, were used in the analysis to identify patterns between these genomes that might be responsible for the variability in biological activity between strains. These comparisons are summarized in the heat map shown in Fig 6.

In this heat map, red and black blocks represent the presence and absence, respectively, of a cluster member in a given genome. Surprisingly, the hierarchical clustering of these strains by protein sequence similarity suggested that SCB34 appears to have a functionally different protein coding capacity than strain RS218. RS218 showed close similarity to *E*. *coli* intestinal isolates SE15, a commensal strain isolated from the feces of a healthy adult [26], and to NRG857c, an adherent-invasive O83:H1 *E*. *coli* recovered from a patient with Crohn’s disease [27]. Strain SCB34, on the other hand, appears to be most similar to a small clade of strains that includes *E*. *coli* CFT073, a prototypic extra-intestinal pathogenic *E*. *coli* (ExPEC) isolate recovered from a bacteremic woman with pyelonephritis [28], and to *E*. *coli* 07798, an O157:H39 atypical enteropathogenic (aEPEC) strain isolated from a patient with diarrhea [29]. Among the protein coding sequences that are shared by SCB34, CFT073, and 07798, and that show no similarity to RS218, are some proteins encoded by a *fim* gene cluster, which encode elements of type 1 fimbriae. The FimF protein in this cluster (accession number KDN05313.1) shows 98% similarity to corresponding coding sequences in CFT073 and 07798, and no similar sequence in RS218. In addition, FimD (accession number KDN05314.1) in SCB34 shows 99% similarity with sequences present in both CFT073 and 07798, but these sequences share less than 67% similarity to those encoding this protein in RS218. Another protein coding sequence shared among SCB34, CFT073, and 07798 is sequence KDN05904.1, which is 98% similar to antitoxin HigA present in both CFT073 and 07798. This toxin-antitoxin system is not found in RS218. Furthermore, SCB34 encodes a serine protease autotransporter enterotoxin EspC (accession number KDN06139.1), whose beta-barrel domain is similar to the C-terminus domain of the Pic serine protease precursor also present in CFT073 (WP_001045652.1), and to the C-terminus domain of the SepA autotransporter encoded by 07798 (EKI44969.1) (S1 Fig). This domain is only 67% similar to a sequence annotated as a “disrupted peptidase” present in RS218.

In addition to identifying the above similarities between SCB34 and representative *E*. *coli* genomes, the CD-hit analysis also suggested that 282 protein coding genes that were unique to SCB34 (S2 Table). These 282 unique genes include 147 genes that encode hypothetical proteins, and 135 genes with known function. Several of these genes identified as unique based on the thresholds used by CD-hit (80% identity, covering 80% of the sequence), were closely similar to sequences in some of the comparison strains included, e.g., some showed 100% identity to <80% sequence coverage. Therefore, manual curation of the CD-hit generated list was performed using BLASTn as described in Methods section in order to identify the subset of sequences with zero identity to those in strains included in our initial comparison. This additional analysis yielded 58 of such sequences which are presented in S3 Table. Among these 58 SCB34 unique genes with known function we found those encoding a L-lactate permease (KDN05013.1), and a zinc peptidase (KDN08838.1). Another unique sequence in SCB34 encodes an altronate hydrolase, a component of the pathway for the degradation of hexuronates, which are utilized by some intestinal *E*. *coli* strains during growth in intestinal mucus [30]. Additional unique protein coding sequences found in SCB34 are those of an O-antigen synthesis cluster that is not present in any strain included in our comparisons. This region encompasses sequences highly similar to those encoding O25b antigens in ST131 strains [31], and includes a dTDP-4-dehydrorhamnose 3, 5-epimerase, several glycosyltransferases, and a Wxz flippase involved in the membrane translocation of O-antigen. We also identified a transcriptional regulator of the Ic1R family (KDN04846.1).

In order to confirm that our analysis reliably identifies unique factors relevant in various *E*. *coli* pathotypes, we performed an additional CD-hit comparison that focused on 16 strains among our initial group of 35 for which experimental data exist that confirm the role of some of their individual virulence factors in determining their pathotype. We also included in this additional comparison three of the commensal strains and the laboratory strain selected in our initial analysis (Table 1). This analysis showed that the number of unique genes in each of these 16 strains, when compared to the initial group of 35, is variable. For example, we found 197 and 846 unique genes in each of the AIEC we included, respectively, and a range from 135 to 544 unique genes in UPEC strains. Commensal strains showed a range from 73 to 370 unique genes. The lowest number of unique genes was found in laboratory strain MG1655.

**Table 1:** Representative pathotypes and number of unique genes in 16 strains selected among all strains included in Fig 6.

*E*. *coli* Strain	Pathotype	Unique genes (n)
NRG857c	AIEC	197
541–15	AIEC	846
O104:H4 str. 2011C-3493	EAEC	236
Sakai	EHEC	489
48	EHEC	560
E2348/69	EPEC	288
07798	aEPEC	545
ETEC H10407	ETEC	252
CFT073	UPEC	544
IAI39	UPEC	135
UMN026	UPEC	212
KTE33	UPEC	504
ED1a	Commensal	370
SE15	Commensal	73
SE11	Commensal	294
MG1655	Laboratory strain	68

AIEC, Adherent invasive *E*. *coli*; EAEC, enteroaggregative *E*. *coli*; EHEC, enterohemorrhagic *E*. *coli*; EPEC, enteropathogenic *E*. *coli;* aEPEC, atypical enteropathogenic *E*. *coli*; ETEC, enterotoxigenic *E*. *coli*; UPEC, uropathogenic *E*. *coli*.

Although the total number of unique genes in each of these 16 strains did not consistently associate with a particular pathotype, this analysis confirmed that our comparisons can distinguish individual virulence factors that are known to be characteristic of specific pathotypes. Examples of this include the presence of unique genes encoding colicins in strain NRG857c, type II and III secretions system proteins in strain Sakai, *agg* and *aat* genes in strain O104:H4 str. 2011C-3493, *espC* and *efa1* in strain E2348/69, *tibA* in strain ETEC H10407, and unique enterobactin synthesis genes in CFT073. S5 Table contains all the unique protein coding sequences found by CD-hit comparisons in each of these 16 strains compared to the larger group of 35 *E*. *coli* strains.

To further understand the phenotypic differences between SCB34 and RS218, we performed an additional analysis to identify unique genes in these two invasive neonatal isolates, compared to the focused group of 16 strains. The CD-hit comparison among these 18 strains demonstrated 471 unique genes in SCB34 and 206 in RS218, thus corroborating the greater complexity of the SCB34 compared to RS218. Moreover, we also confirmed the presence of unique protein coding sequences in RS218 such as those encoding three unique glycotransferases likely involved in the synthesis of O antigen, a fimbrial protein (AJM73146.1), and several membrane proteins including AJM73951.1, AJM73684.1, and AJM73683.1. The unique SCB34 and RS218 genes obtained from the CD-hit comparison are presented in S6 Table.

**Fig 7:**
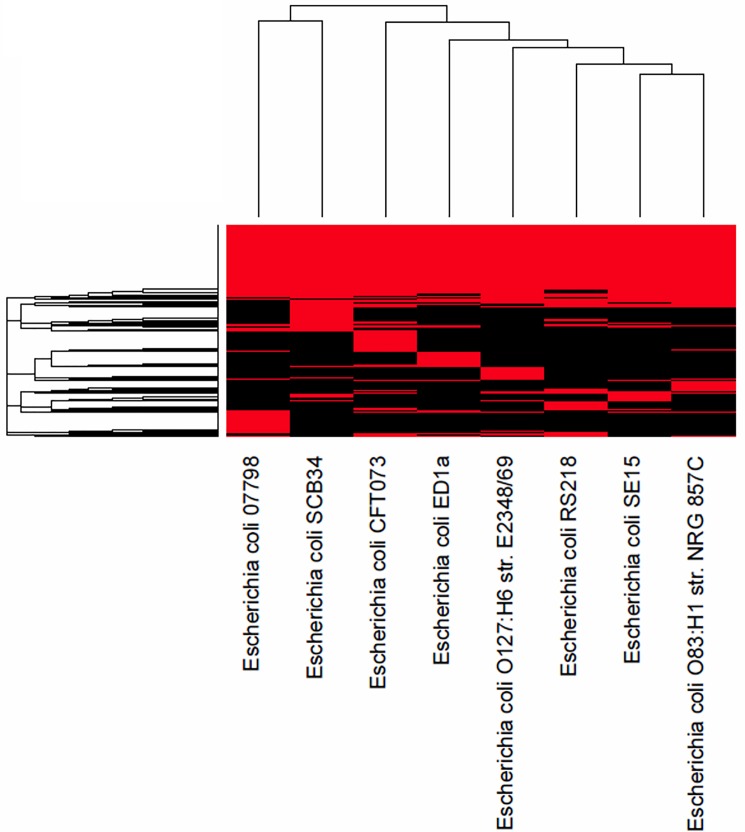
Heatmap demonstrating the relationship of *E*. *coli* strains belonging to phylogroup B2, including SCB34 and RS218.

SCB34 and RS218 belong to phylogroup B2, which is overrepresented among *E*. *coli* isolates producing extra-intestinal infections. However, despite sharing the same phylogroup, these two neonatal isolates possess unique genotypic characteristics that are likely to determine their individual phenotypes and that distinguish them from each other. This is highlighted in the heatmap shown in Fig 7 which shows a comparison of only the B2 strains among those in Table 1.

## Discussion

In this study we have demonstrated that both SCB34 and RS218, two *E*. *coli* strains that produced neonatal bacteremia in humans, possess a high invasion ability into intestinal epithelial cells *in vitro*, and cause septicemia after oral acquisition in newborn animals. *E*. *coli* strains belonging to different pathotypes, and other invasive Gram-negative bacteria such as *Shigella* and *Salmonella* have shown variable invasion rates of epithelial cells [32, 33]. Our *in vitro* invasion studies produced comparable results to previous experiments that evaluated the invasion ability of T84 epithelial cells by RS218 and demonstrated a significantly greater invasion by RS218 compared to the nonpathogenic HB101 strain [9].

Other researchers have shown that neonatal invasive *E*. *coli* strains produce septicemia in newborn animals after oral inoculation [7, 8, 10, 34–37]. Most prior studies have investigated the virulence of neonatal *E*. *coli* isolates after oral inoculation in newborn animals using strains that express the K1 capsule, likely because 70–90% of neonatal meningitis *E*. *coli* isolates are known to possess this virulence factor [38–40]. However, only 30–60% of *E*. *coli* strains that cause bacteremia without meningitis in young infants carry the K1 capsule [15, 38, 41–43]. Although the K1 capsule plays a significant role in the development of (particularly high-degree) bacteremia, its role is not essential for the development of neonatal sepsis after bacterial oral acquisition as we demonstrated in our experiments performed with SCB34, a non-K1 *E*. *coli* neonatal isolate. The K1 capsule and other virulence factors have been identified as relevant in the pathogenesis of neonatal meningitis [13], but the factors that facilitate intestinal invasion and transcytosis of neonatal *E*. *coli* isolates have not been fully ascertained. SCB34, a non-K1, contemporary ST131 *E*. *coli* clinical isolate produced bacteremia in newborn animals when administered orally but did not after IP injection in our experimental model. In contrast, RS218 produced neonatal bacteremia when administered *via* either route. This phenomenon could be explained by the presence of virulence factors in SCB34 that determine its lethal phenotype only when acquired orally, and that are dependent on their interaction with the neonatal gut to render this organism fully virulent. Thus, the particular virulence phenotype of SCB34 provides an opportunity to expand our understanding of the mechanisms that determine the development of neonatal bacteremia after oral acquisition of *E*. *coli*.

Both SCB34 and RS218, belong to phylogroup B2 which is largely represented among *E*. *coli* strains that cause bacteremia in newborns [42, 44]. We speculate that specific virulence factors relevant to the pathogenesis of neonatal bacteremia are present in phylogroup B2 *E*. *coli* strains that can cause disease through various mechanisms, and that despite the phenotypic heterogeneity among these isolates, the phylogenetic signal of these strains is not obscured. Phylogroup assignment is thus relevant for the epidemiological characterization of neonatal invasive *E*. *coli* strains. However, whole-genome sequencing data provides more precise information of the content and variation of specific virulence factors involved in the pathogenic mechanisms utilized by these strains [45].

Only a few virulence factors have been demonstrated in neonatal invasive *E*. *coli* strains to be relevant in the bacterial interaction with intestinal epithelium [46–49]. Oral inoculation with mutants lacking *clbA* and *clbP*, which are genes involved in colibactin synthesis and maturation, respectively, resulted in decreased intestinal colonization and virulence in newborn animals [37]. These genes are within the *pks* 54-kb genomic island present in many ExPEC strains with high virulence characteristics [50]. Interestingly, SCB34 lacks *pks*-associated genes, including *clbA* and *clbP*. This highlights the existence of additional unknown virulence factors that are present in neonatal invasive *E*. *coli* strains such as SCB34, that are critical in the process of intestinal translocation that precedes neonatal septicemia. Moreover, and in contrast to RS218, SCB34 does not carry a plasmid [16]. The 114 kb plasmid in RS218 encodes several virulence factors that are relevant to the *in vitro* invasion properties of this strain, and to its ability to cause septicemia and meningitis in newborn rats [51]. In SCB34, all the necessary virulence factors to cause septicemia after oral infection are on its chromosome.

The cluster analysis revealed that each strain possesses protein compositions that can delineate them from one another, and clustered them with other representative *E*. *coli* strains with similar proteomes. The distinct genomic characteristics between RS218 and SCB34 likely determine the phenotypic profile that we observed in our experiments. For example, RS218 carries the *neuDBACES* genes specific for the K1 capsule, a well-known factor that interferes with complement-mediated killing and phagocytosis [52]. These genes are not present in SCB34, or in any of the strains we included in our comparisons, with the exception of IAI39. This trait would explain at least in part, the ability of RS218 to be pathogenic when injected IP which is contrast to the phenotype that we observed in SCB34.

We found that RS218 was most similar to *E*. *coli* isolates SE15 and NRG857c. SCB34, on the other hand, was most similar to *E*. *coli* strains 07798 and CFT073. Among the virulence factors shared by SCB34 with CFT073 and 07798, but not with RS218, we found components of type 1 fimbriae, which are known to be relevant in the pathogenesis of urinary tract infections by contributing to bacterial colonization and invasion of uroepithelium, biofilm formation, and establishment of intracellular bacterial communities [53, 54]. The distribution of various fimbrial gene clusters among *E*. *coli* is characteristic of particular pathotypes [55]. FimF, for example, is a tip fibrillum component that functions as an adaptor and is involved in the adhesive properties of these structures [56]. FimF is relevant to the epithelial invasion process by *E*. *coli* [57] but its role in the pathogenesis of neonatal bacteremia caused by *E*. *coli* intestinal invasion and transcytosis in the neonatal gut has not been yet investigated.

Another protein-coding sequence similar among these three strains that is absent in RS218 is the antitoxin HigA, a component of the HigB-HigA type II toxin-antitoxin module ubiquitously present in bacteria [58]. The *higBA* loci contribute to bacterial adaptation by the generation of persisters, individual cells that have entered a dormant state that renders them tolerant to antibiotics and other environmental stressors [59]. Sequences encoding the beta domain of an EspC protein in SCB34 were also shared with CFT073, and 07798 but not with RS218. EspC is secreted by the T5SS and internalized by the T3SS into intestinal epithelial cells where it causes cytoskeletal damage by cleaving cytoskeleton and focal adhesion proteins [60]. Although the effector domain was not shared among SCB34, CFT073, and 07798, the common beta-barrel structure in these three strains may be a relevant factor by serving as a translocation domain for various effectors possibly relevant to virulence in these strains, as the nonspecificity of the beta domain to a single passenger domain has been suggested [61] Whether these, or other shared virulence factors present in SCB34 could be relevant to the pathogenesis of *E*. *coli* strains causing neonatal sepsis remains to be investigated.

Our initial CD-hit analysis also identified 282 unique coding sequences in SCB34, and from this group, manual BLASTn inspection yielded 58 sequences that are absent entirely in all of the representative strains studied. Another unique sequence in SB34 encodes an altronate hydrolase. Altronate hydrolases degrade D-galacturonates, which are present in intestinal mucus and are a nutritional source for pathogenic *E*. *coli* strains. Although this degradation pathway does not appear to be directly involved in intestinal colonization [62], it does confer a selective growth advantage in the mammalian intestine [63]. Moreover, genes involved in the metabolism of D-galacturonate were upregulated during growth of pathogenic *E*. *coli* in urine, and human blood [64, 65]. The direct role of this metabolic pathway in the pathogenesis of neonatal *E*. *coli* sepsis has not been investigated. SCB34 is also unique compared to the strains used in this study in regards to the presence of the O25b antigen, a characteristic common to ST131 strains that have recently disseminated in populations of all ages worldwide, and are known for carrying multiple antibiotics resistance and virulence factor genes [66]. The prevalence of ST131 strains as a cause of invasive disease in newborns and infants continues to increase for reasons that are unclear [15, 67]. It has been proposed that ST131-O25b strains have an enhanced ability to colonize the gut [68]. Colonized hosts by these strains include children in day care who may contribute as a reservoir [69]. Specific O antigens are known to contribute to *E*. *coli* virulence by enhancing the bacterial interaction with epithelial barriers. The O6 antigen of CFT073 promotes colonization of the urinary tract by this strain [70]. Moreover, restoration of the O16 antigen in strain MG1655 enabled it to colonize the gut and produce a lethal infection in *Caenorhabditis elegans* [71]. Whether the O25b antigen present in ST131 strains such as SCB34 specifically enhances the ability of neonatal *E*. *coli* strains to colonize, invade and transcytose intestinal epithelium will need to be elucidated. Among the unique proteins in SCB34 we also identified an Ic1R transcriptional regulator. The Ic1R family of regulators controls carbon metabolism and plant virulence in certain enterobacteriaceae, multidrug resistance, solvent tolerance in *Pseudomonas*, and inactivation of quorum sensing signals in *Agrobacterium* [72]. It is possible that similar mechanisms controlled by this regulator in SCB34 also play a role in the pathogenesis of intestinal invasion and virulence by this isolate in newborns. Another unique protein we found in SCB34 is YjbH, an outer membrane lipoprotein involved in exopolysaccharide synthesis and predicted to have a β-barrel conformation [73]. Exopolysaccharides in *E*. *coli* are relevant to its virulence because they are involved in cell attachment, biofilm formation, and protection against host innate immune responses. The role of this unique protein as a relevant virulence factor to the pathogenesis of SCB34 in newborns will need to be investigated.

Our project has identified unique protein coding sequences in SCB34, a neonatal bacteremia isolate that requires the interaction with the newborn’s gut to produce septicemia. These finding are the basis for additional *in vitro* and *in vivo* investigations currently undergoing in our laboratory, aimed at elucidating the role of the unique factors in SCB34 that are relevant to the pathogenesis of neonatal *E*. *coli* septicemia.

## Conclusions

In summary, we have demonstrated that the *E*. *coli* clinical isolate SCB34 has the distinct ability to produce neonatal bacteremia after oral inoculation but not after IP injection, and to invade and transcytose intestinal epithelial cells more efficiently as compared to the archetypal neonatal meningitis isolate RS218. The phenotypic characteristics of SCB34, along with the details of its unique gene repertoire revealed in this study, highlight the existence of several virulence factors that could participate in the development of septicemia after *E*. *coli* oral acquisition in newborns. In addition to the known virulence factors unique to SCB34, our study identified 147 hypothetical proteins with a potential role in the pathogenesis of neonatal *E*. *coli* bacteremia. The characterization of these factors is underway in our laboratory. Our results provide the foundation for an improved understanding of the pathogenesis of neonatal sepsis, and thus for the development of novel strategies against bacteremia caused by oral route of infection with *E*. *coli* in newborns.

## Supporting information

S1 Table Orthologous clusters found among 35 *E*. *coli* strains, and corresponding accession numbers. (XLSX)

S2 Table SCB34-unique protein coding sequences identified by CD-hit comparison of orthologous sequences with representative *E*. *coli* strains. (XLSX)

S3 Table SCB34-unique protein coding sequences with zero identity to sequences in representative *E*. *coli* strains selected by manual curation from initial CD-hit output shown in S2 Table. (DOCX)

S4 Table *E*. *coli* strains included for comparison with neonatal *E*. *coli* isolates SCB34 and RS218. (DOCX)

S5 Table Unique protein coding sequences present in each of 16 strains compared to our initial larger group of 35 *E*. *coli* strains. (XLS)

S6 Table Unique protein coding sequences present in SCB34 and RS218 compared to our focused 16-strain analysis. (XLS)

S7 Table Results of bacteremia in surviving pups after oral or intraperitoneal inoculation. (DOCX)

S1 Fig Schematic representation of the EspC-like homologous regions in SCB34, CFT073, and 07798. Comparisons between SCB34 (sequence in the middle), CFT073 (top sequence), and 07798 (bottom sequence) were performed by employing the ACT software tool (Sanger Institute, http://www.sanger.ac.uk). NCBI reference sequence and GenBank numbers are indicated at the left of each sequence.
(TIF)
