# Supplementary material for: Route of infection alters virulence of neonatal septicemia Escherichia coli clinical isolates
Source: PLoS One. 2017 Dec 13;12(12):e0189032. doi: 10.1371/journal.pone.0189032 (PMC5728477; doi:10.1371/journal.pone.0189032)
Supplement: S3 Table — (DOCX) [file pone.0189032.s003.docx]

**S3 Table. SCB34-unique protein coding sequences with no identity to sequences in representative *E. coli* strains selected by manual curation from initial CD-hit output shown in S2 Table.**

| **Accession** | Annotation |
| --- | --- |
| KDN04616.1 | Type I restriction-modification system |
| KDN04843.1 | 4-hydroxythreonine-4-phosphate dehydrogenase |
| KDN04844.1 | Altronate hydrolase |
| KDN04845.1 | hypothetical protein DH22_4346 |
| KDN04846.1 | Transcriptional regulator |
| KDN05004.1 | hypothetical protein DH22_4336 |
| KDN05005.1 | hypothetical protein DH22_4337 |
| KDN05006.1 | putative kinase |
| KDN05013.1 | L-lactate permease |
| KDN05116.1 | hypothetical protein DH22_4220 |
| KDN05117.1 | hypothetical protein DH22_4221 |
| KDN05119.1 | hypothetical protein DH22_4223 |
| KDN05298.1 | hypothetical protein DH22_4214 |
| KDN05725.1 | hypothetical protein DH22_3582 |
| KDN06144.1 | hypothetical protein DH22_3091 |
| KDN06145.1 | hypothetical protein DH22_3092 |
| KDN06146.1 | hypothetical protein DH22_3093 |
| KDN06316.1 | hypothetical protein DH22_2995 |
| KDN06321.1 | hypothetical protein DH22_3000 |
| KDN06556.1 | hypothetical protein DH22_2890 |
| KDN06559.1 | hypothetical protein DH22_2893 |
| KDN06798.1 | transposase |
| KDN06799.1 | Aminoglycoside N(3')-acetyltransferase III (ACC(3)-III) (Aminocyclitol 3-N-acetyltransferase type III) (Gentamicin-(3)-N-acetyl-transferase) |
| KDN06899.1 | Membrane protein involved in the export of O-antigen |
| KDN06900.1 | Glycosyltransferase |
| KDN06901.1 | Glycosyltransferase |
| KDN06902.1 | hypothetical protein DH22_2414 |
| KDN06903.1 | O-acetyltransferase |
| KDN06904.1 | putative glucose transferase |
| KDN06905.1 | glycosyl transferase |
| KDN06929.1 | hypothetical protein DH22_2441 |
| KDN06932.1 | hypothetical protein DH22_2444 |
| KDN06940.1 | hypothetical protein DH22_2452 |
| KDN06944.1 | cII |
| KDN06974.1 | hypothetical protein DH22_2486 |
| KDN07009.1 | hypothetical protein DH22_2295 |
| KDN07014.1 | hypothetical protein DH22_2300 |
| KDN07021.1 | hypothetical protein DH22_2307 |
| KDN07022.1 | hypothetical protein DH22_2308 |
| KDN08133.1 | hypothetical protein DH22_1127 |
| KDN08134.1 | DNA-cytosine methyltransferase |
| KDN08150.1 | hypothetical protein DH22_1144 |
| KDN08262.1 | hypothetical protein DH22_1016 |
| KDN08633.1 | hypothetical protein DH22_0640 |
| KDN08805.1 | hypothetical protein DH22_0401 |
| KDN08811.1 | hypothetical protein DH22_0407 |
| KDN08837.1 | hypothetical protein DH22_0433 |
| KDN08838.1 | Zn peptidase |
| KDN08839.1 | hypothetical protein DH22_0435 |
| KDN08840.1 | hypothetical protein DH22_0436 |
| KDN08845.1 | hypothetical protein DH22_0441 |
| KDN08846.1 | hypothetical protein DH22_0442 |
| KDN08847.1 | hypothetical protein DH22_0443 |
| KDN08848.1 | Replicative DNA helicase |
| KDN08849.1 | hypothetical protein DH22_0445 |
| KDN08850.1 | Adenylosuccinate synthase |
| KDN08852.1 | hypothetical protein DH22_0448 |
| KDN08857.1 | Integrase |
