# Supplementary material for: Route of infection alters virulence of neonatal septicemia Escherichia coli clinical isolates
Source: PLoS One. 2017 Dec 13;12(12):e0189032. doi: 10.1371/journal.pone.0189032 (PMC5728477; doi:10.1371/journal.pone.0189032)
Supplement: S4 Table — (DOCX) [file pone.0189032.s004.docx]

**S4 Table.** *E. coli* strains used for comparison with neonatal *E. coli* isolatesSCB34 and RS218

| ***E. coli* Strain** | **Phenotype** |
| --- | --- |
| SCB34 | Neonatal bacteremia |
| RS218 | Neonatal meningitis |
| NRG857c | AIEC |
| 541-15 | AIEC |
| O104:H4 str. 2011C-3493 | EAEC |
| Sakai | EHEC |
| O111:H-str.11128 | EHEC |
| 0.1288 | EHEC |
| 48 | EHEC |
| 53638 | EIEC |
| E2348/69 | EPEC |
| N1 | EPEC |
| 07798 | aEPEC |
| O139:H28 str.E24377A | ETEC |
| ETEC H10407 | ETEC |
| CFT073 | UPEC |
| IAI39 | UPEC |
| UMN026 | UPEC |
| KTE33 | UPEC |
| KTE 102 | UPEC |
| ED1a | Commensal |
| SE15 | Commensal |
| MS 196-1 | Commensal |
| SE11 | Commensal |
| B str. REL606 | Commensal |
| 2-222-05_S3_C2 | Commensal |
| 1-182-04_S3_C2 | Commensal |
| 1-250-04_S3_C1 | Commensal |
| SC D2 | Commensal |
| H299 | Commensal |
| M718 | Commensal |
| M863 | Commensal |
| B354 | Commensal |
| B185 | Commensal |
| MG1655 | Laboratory strain |

AIEC, Adherent invasive *E. coli*; EAEC, enteroaggregative *E. coli*; EHEC enterohemorrhagic *E. coli*; EIEC, enteroinvasive *E. coli*; EPEC, enteropathogenic *E. coli*; aEPEC, atypical enteropathogenic *E. coli*; ETEC, enterotoxigenic *E. coli*; UPEC, uropathogenic *E. coli.*
