# Supplementary material for: Route of infection alters virulence of neonatal septicemia Escherichia coli clinical isolates
Source: PLoS One. 2017 Dec 13;12(12):e0189032. doi: 10.1371/journal.pone.0189032 (PMC5728477; doi:10.1371/journal.pone.0189032)
Supplement: S7 Table — (DOCX) [file pone.0189032.s007.docx]

**S7 Table**. Results of bacteremia in surviving pups after oral or intraperitoneal inoculation

| *E. coli* Strain | Bacteremic surviving pups/total surviving pups at the end of each experiment protocol | |
| --- | --- | --- |
|  | Oral inoculation | IP inoculation |
| SCB34 | 3/22 | 1/20 |
| RS218 | 7/18 | NA |
| DH5α | 0/30 | 5/20 |

NA, not applicable.
