# Supplementary figures and images for: Route of infection alters virulence of neonatal septicemia Escherichia coli clinical isolates
Source: PLoS One. 2017 Dec 13;12(12):e0189032. doi: 10.1371/journal.pone.0189032 (PMC5728477; doi:10.1371/journal.pone.0189032)

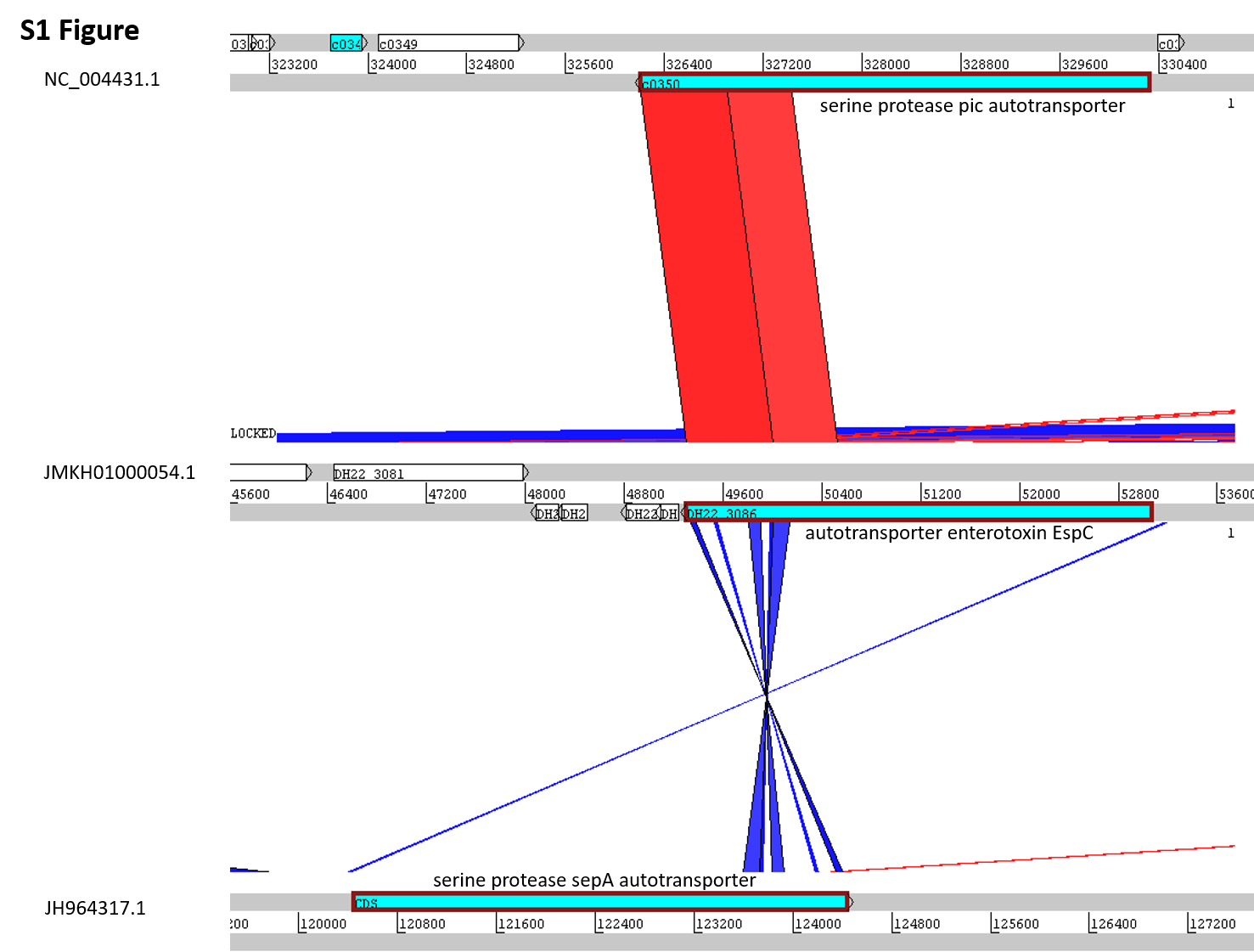

Supplement: S1 Fig — Comparisons between SCB34 (sequence in the middle), CFT073 (top sequence), and 07798 (bottom sequence) were performed by employing the ACT software tool (Sanger Institute, http://www.sanger.ac.uk). NCBI reference sequence and GenBank numbers are indicated at the left of each sequence. (TIF) [file pone.0189032.s008.tif]
